# Fibromyalgia and Depression: A Literature Review of Their Shared Aspects

**DOI:** 10.7759/cureus.24909

**Published:** 2022-05-11

**Authors:** Daniela Yepez, Xavier A Grandes, Ramya Talanki Manjunatha, Salma Habib, Sai Lahari Sangaraju

**Affiliations:** 1 Faculty of Medicine, Universidad Catolica de Santiago de Guayaquil, Guayaquil, ECU; 2 Research, Universidad Catolica de Santiago de Guayaquil, Guayaquil, ECU; 3 Graduate Medical Education, Kempegowda Institute of Medical Sciences, Bangalore, IND; 4 Medicine and Surgery, Institute of Applied Health Sciences (IAHS), Chittagong, BGD; 5 Research, P.E.S. Institute of Medical Sciences and Research, Kuppam, IND

**Keywords:** fibromyalgia pathophysiology, depressive symptoms in fibromyalgia, depression, fibromyalgia and depression, fibromyalgia

## Abstract

Fibromyalgia (FM) is a chronic pain syndrome characterized by widespread, persistent pain that lasts more than three months without an evident organic lesion. FM has been considered controversial throughout history due to its validity as a diagnosis being constantly in question. Most patients diagnosed with FM are females. FM has been associated with multiple conditions, including irritable bowel and psychiatric disorders. Among all associated conditions, depression has been frequently found in patients with FM. Studies suggest that depression negatively affects the outcome of patients with FM. Moreover, a bidirectional relation between FM and depression has been depicted: depression increases the risk of FM being diagnosed later in life, as well as FM increases the risk of developing depression. In this article, we discussed aspects that FM and depression share and that might link both diseases, such as certain elements they seem to share in their pathophysiology: predisposing and triggering factors, central sensitization and kindling, areas of the brain implicated in both pain modulation and mood regulation, and hypothalamic-pituitary-adrenal axis (HPA axis) alterations. In addition, we highlighted the prevalence of depression in patients with FM, overlapping symptoms between FM and depression and how to assess them, and treatment strategies that have shown effective management of both conditions when concomitant. Due to the improvement of many aspects of FM when depression is appropriately targeted, screening for depression in patients with FM, despite its difficulty, has been encouraged.

## Introduction and background

Fibromyalgia (FM) has been defined as a chronic pain syndrome whose main characteristic is widespread chronic pain that lasts more than three months in the absence of an evident organic lesion [1]. FM has always been considered a controversial condition, with ongoing wars concerning the validity of the diagnosis [2-4]. Not too long ago, in 1990, the American College of Rheumatology (ACR) acknowledged FM as a “long known but generally neglected syndrome” and provided a consensus definition for FM as well as, for the first time, rigorously tested diagnostic criteria [5]. The prevalence of FM in the general population is approximately 1 to 5 %, with female predominance, and it is also more prevalent in patients over 50 years old [6,7]. Pathogenesis of FM involves both genetic and environmental factors. Genetic factors that might predispose to FM include polymorphisms of the D4 dopamine receptor gene and the adrenergic receptor gene [8,9]. Other genes found to be involved in FM susceptibility include: trace amine-associated receptor 1 (TAAR1), regulator of G-protein signaling 4 (RGS4), cannabinoid receptor 1 (CNR1), glutamate receptor, ionotropic, and alpha-amino-3-hydroxy-5-methyl-4-isoxazole propionate (AMPA 4) (GRIA4) [10]. Moreover, the genetic contribution to the etiology of FM is supported by its strong familial aggregation [11]. Environmental factors that have been associated with FM and that can precipitate it include: sexual assault/abuse, physical assault/abuse, and childhood emotional abuse and neglect [12,13].

Regarding the pathophysiology of FM, many mechanisms have been proposed and studied. FM is related to dysfunction in neurotransmitters, mainly in mono-aminergic neurotransmission, which leads to increased levels of excitatory neurotransmitters (substance P, glutamate), along with decreased levels of norepinephrine and serotonin at the level of descending anti-nociceptive pathways in the spinal cord [14]. Both peripheral and central sensitization coexist in FM, being responsible for the pain and hypersensitivity seen in patients with FM [15,16]. Peripheral sensitization also induces and maintains central sensitization [15]. The latter accounts for the response to inputs that should be ineffective under normal conditions, allowing greater perception of pain [16]. Other findings related to FM pathophysiology include: alterations of cortisol circadian rhythm and proinflammatory cytokine involvement [14,17,18]. The criteria for FM, revised in 2016, establish the diagnosis of FM in patients that satisfy the following conditions: widespread pain index (WPI) greater than or equal to 7 and a severity scale score (SSS) greater than or equal to 5, or WPI of 4-6 and SSS greater than or equal to 9; the presence of pain in at least four of five regions (generalized pain); persistence of symptoms for at least three months [19]. FM has been associated with various somatic pain conditions, psychiatric conditions, sleep disorders, and rheumatic diseases [20]. These associated conditions include: irritable bowel syndrome (IBS), temporomandibular disorders, chronic headaches and migraine, mood disorders, and depression [21-24]. Management of FM involves non-pharmacological and pharmacological interventions, with the education of the patient regarding the nature of their disease considered to be the first step [25]. Then, non-pharmacological approaches should be integrated. Physical exercise adjusted to the patient’s functional level is strongly recommended and can be paired with other therapies such as acupuncture or hydrotherapy. Individualized treatment should be considered for every patient’s specific need, such as pharmacotherapy for severe pain or severe sleep problems (tramadol, gabapentin, duloxetine, milnacipran, among others) and cognitive behavioral therapy (CBT) for pain management and concomitant depression or anxiety [26].

Depression is one of the main causes of disability worldwide, and it is also considered a major contributor to the overall global burden of disease [27]. Between FM and depression, a bidirectional association has been found: depression increases the risk of FM being diagnosed later in life, and FM increases the risk of developing depression [28]. Patients with persistent pain are more likely to meet the diagnosis criteria of depression compared to those without pain [29]. In addition, the odds of developing depression are higher for patients presenting multiple pain sites than for those with fewer pain sites [30]. Given the impact that both FM and depression have on patients’ quality of life, it is essential to diagnose depression and treat it along with FM properly. This review article aims to highlight the prevalence of depression among patients diagnosed with FM and explores the underlying aspects that both diseases seem to share.

## Review

Intertwined pathophysiology

**Figure 1:**
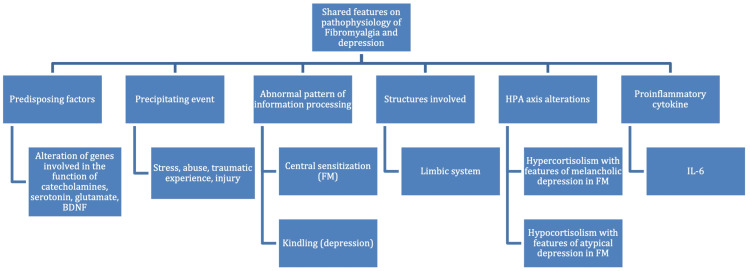
Summary of the shared features on the pathophysiology of FM and depression. CRF: corticotropin-releasing factor; BDNF: brain-derived neurotrophic factor; FM: fibromyalgia; HPA axis: hypothalamic–pituitary–adrenal axis; IL-6: interleukin 6. Image credit: Daniela Yepez.

Genetic predisposing factors have been well studied for FM and depression. Precipitating events can trigger FM in susceptible patients [12,13]. As for depression, the genetic alterations associated do not cause this disorder per se but increase the risk of developing depression as a response to a precipitating event [31]. These genes participate in the function of catecholamines, serotonin, corticotropin-releasing factor, monoamines, glutamate, and brain-derived neurotrophic factor [32]. A polymorphism in the serotonin transporter (5-HTT) gene, involved in major depressive disorder (MDD), appears to be implicated in FM as well [33]. Sharing remarkable similarity to the central sensitization implicated in FM, the “kindling hypothesis” has been suggested as a mechanism that occurs in MDD. The kindling hypothesis states that, due to an abnormal pattern of information processing, each episode of depression increases the likelihood of a subsequent new episode of depression that will have less influence from environmental adversity than the first episode had [16,34]. It is even suggested that sensitization and kindling could share neurobiological bases, such as alterations in gene expression and neuroplastic changes [35]. The areas of the forebrain implicated in pain modulation include the limbic system: amygdala, hippocampus, hypothalamus, as well as insular and anterior cingulate cortical regions. Elements from this circuit also participate in regulating stress response and mood [36]. Ross et al. found that, among MDD subtypes, atypical and melancholic depression are the most prevalent subtypes in patients with FM. In addition, patients with FM and atypical or melancholic depressive features presented increased severity on all clinical features compared to patients without depression [37]. Furthermore, Gold et al. observe that both subtypes of MDD are associated with alterations of the HPA axis: melancholic depression has been associated with hypercortisolism, whereas atypical depression seems related to hypocortisolism. They also suggest that, in FM, a progression might be occurring: the early stage of FM is associated with hypercortisolism and melancholic depression, and as the disease progresses, the cortisol response drops below normal levels, which leads to hypocortisolism and manifests atypical depression features [38]. Microglial activation, therefore, neuroinflammation in CNS, has been found in both FM and depression [39,40]. Both FM and depression can be precipitated by stress in the form of injury, and traumatic experience, among others [41,42]. FM is associated with increased serum concentrations of proinflammatory cytokine interleukin (IL)-6 and IL-8. Elevated IL-6 and IL-8 correlate with increased scores on the fibromyalgia impact questionnaire (FIQ), suggesting they have a role in the persistent pain observed in patients with FM [43]. IL‐6 is associated with fatigue, stress, hyperalgesia, and depression, while IL-8 mediates sympathetic hyperalgesia [44,45]. Increased serum concentrations of IL-6 are also observed in depression, and this finding is associated with increased severity of depressive symptoms in patients that do not respond to antidepressants [46,47]. On the other hand, a relation between IL-8 and depression has not been firmly established [48]. A summary of the factors mentioned above is presented in Figure 1.

Evidence on the prevalence of depression in fibromyalgia

Kleykamp et al. estimated in their meta-analysis the prevalence of multiple psychiatric and chronic pain comorbidities in patients with FM. Their meta-analysis included, for the estimation of depression and MDD, studies in which the diagnosis of psychiatric disorders was established through in-person assessments performed by mental health professionals, applying standardized assessment tools. The studies selected were cross-sectional, reporting estimated prevalence, and published between 1992 and 2018. The sample size for all the studies ranged from 22 to 509, where most of the participants were female patients (80% or more). They concluded that the most prevalent comorbidity in FM was depression (not specified) and MDD in FM, with a current prevalence for depression (not specified) and MDD of 43.0% and 32.0%, respectively. In addition, the estimated lifetime prevalence for the disorders abovementioned was 63.0% and 52.3%, respectively [49].

Loge-Hagen et al. found similar results in 2018. Their meta-analysis included 11 studies, which featured sample sizes larger than 30 individuals. The participants were adults (more than 18 years old), and none of them presented another rheumatologic comorbidity. All the selected studies were peer-reviewed. Almost all the selected studies were cross-sectional, and the total sample size was 1,316. They concluded that one-fourth of all patients with a previous diagnosis of FM presented concomitant MDD, and more than half of them experienced MDD throughout their lives [50].

Singh and Kaul conducted a cross-sectional study in an outpatient clinic in India, with the participation of 80 patients with FM and 72 healthy controls, matched by age and sex. Patients receiving treatment with antidepressants and patients with other comorbidities were excluded. They found a significantly higher prevalence of depression (72.5%) in diagnosed FM patients than in the control group. Their main recommendation was to routinely screen for depression and anxiety in FM patients [51].

Kassam and Patten used data from the Canadian Community Health Survey in 2006 to find the prevalence of MDD in patients that have been diagnosed before with FM, performing a large-scale study with a sample size of 115,160, excluding younger than 18 years old. Results emphasized that the annual prevalence of MDD was three times higher in subjects with FM (22.2%) compared to patients without this condition (7.2%) and that, despite the stratification by sex, age, education, marital status, and household income, this association prevailed in all the groups [52]. A summary of all the studies mentioned above is presented in Table 1.

**Table 1:** Summary of included studies showing the prevalence of depression in patients with FM. MDD: major depressive disorder; FM: fibromyalgia.

References	Design	Sample size	Population	Conclusion
Kleykamp et al. 2020 [49]	Meta-analysis	-	80% of patients were females.	The most prevalent comorbidity in FM is depression (not specified) and MDD.
Loge-Hagen et al. 2019 [50]	Meta-analysis	1,316	FM as the primary diagnosis, no other rheumatologic disease, 18 years old or more.	One-fourth of all patients diagnosed with FM presented with MDD, and more than half of them experienced MDD throughout their lives.
Singh and Kaul 2018 [51]	Cross-sectional	Cases: 80 patients with FM. Controls: 72 healthy individuals.	Groups were matched by sex and age, no current treatment with an antidepressant, no comorbidities.	Higher prevalence of depression (72.5%) in patients with diagnosed FM in comparison to the control group.
Kassam and Patten 2006 [52]	Population-based cross-sectional	115,160	Previously diagnosed with FM, 18 years old and older.	The annual prevalence of MDD was three times higher in FM patients. Prevalence remained high in all the groups.

Struggles in diagnosis: overlapping symptoms

Given that all diagnostic survey instruments used to diagnose depression are at risk of presenting criteria contamination bias due to somatic symptoms of FM (such as chronic pain and fatigue), depression becomes challenging to assess in patients with FM. However, as even subthreshold mood dysfunction can worsen the socioeconomic functioning and pain in patients with FM, thorough screening for depression is needed [53].

Commonly used instruments for measuring and assessing depression do not consider the relationship between pain and depression. They should not be applied to patients with chronic pain, a scenario where they lack sensitivity and criterion contamination occurs [54]. Patients affected by chronic pain tend to manifest problems in areas such as work, sleep, and performance of daily life activities; in addition, they frequently report health concerns, irritability, dissatisfaction, loss of appetite, and fatigue. These symptoms, or items in the test, are more related to pain (somatic aspect) than to mood, which might be the main reason why these patients commonly obtain high scores on rating scales for depression [55]. However, Mood Spectrum Self-Report (MOODS-SR) has been recommended as a suitable instrument for screening FM patients for depression because it allows the recognition of subthreshold mood symptoms while presenting minimal or zero contamination by the somatic aspect of comorbidities [56].

MOOD-SR is designed to assess the lifetime fluctuation between the presence and absence of the complete range of features that can be observed in mood psychopathology. Many of these features are frequently present in clinical populations but are not described in the current psychiatric classification, which mostly mentions the core or criterion symptoms. MOODS-SR comprises 140 yes/no questions, organized into seven symptom domains: mood-depressed, mood-manic, energy-depressed, energy-manic, cognition-depressed, cognition-manic, and rhythmicity and vegetative function [57]. Table 2 summarizes MOODS-SR domains and the aspects they explore.

**Table 2:** Summary of MOODS-SR domains and the explored aspects. MOODS-SR: Mood Spectrum Self-Report Adapted from Dell'Osso et al. [57].

Domain	Subdomains	Explorations
Mood	Mood-depressed	Mood lability and associated changes in interest directed toward family, friends, romantic relationships, work, hobbies, and sports.
	Mood-manic	
Energy	Energy-depressed	Presence of periods of time and situations with a significant change in energy levels.
	Energy-manic	
Cognition	Cognition-depressed	Changes in cognition associated with energy or mood dysregulation.
	Cognition-manic	
Rhythmicity and vegetative functions		Changes in energy, physical well-being, and mental and physical efficiency related to the weather, the seasons, the changes in eating, sleep, and sexual activities.

Integrated management of FM and depression

Pharmacology

Another aspect that seems to link FM and depression is the efficacy of pharmacological treatment with serotonin-norepinephrine reuptake inhibitors (SNRIs) in both conditions [58]. Of the three drugs approved by the US Food and Drug Administration (FDA) for the treatment of FM, two of them belong to this category of antidepressants: duloxetine and milnacipran; the third drug is pregabalin [59]. SNRIs improve both chronic pain and depression, but the effects are relatively independent of each other: improvement in mood is a direct effect of SNRIs, being independent of pain relief; although there is, as expected, an indirect effect over mood thanks to the improvement in pain, and vice versa [60].

Lee and Song performed a meta-analysis comparing the efficacy and tolerability of the three FDA-approved drugs in 2016. They selected nine randomized controlled trials that compared duloxetine, milnacipran, and pregabalin, at recommended doses, with placebo for the treatment of FM. To be included, the studies had to have defined FM according to the American Rheumatology Association criteria and have provided conclusions regarding the clinical efficacy and tolerability of the drugs. The total population of this meta-analysis was 5,140 individuals diagnosed with FM. They concluded that duloxetine is superior to milnacipran and pregabalin for pain improvement [61].

In 2010, Häuser et al. conducted a meta-analysis comparing duloxetine, pregabalin, and milnacipran in terms of efficacy and harm. The studies selected were randomized controlled trials comparing at least two drugs or any of the three drugs with a placebo. The studies had to report outcomes of at least one of the following: pain, fatigue, depressed mood or quality of life, in addition to data regarding adverse effects. All the studies included patients diagnosed with FM as per American Rheumatology Association criteria, 18 years old or more, without any other rheumatic disease. Their meta-analysis reported the superiority of duloxetine compared to pregabalin in improving depressive symptoms in patients with FM [62]. However, two randomized controlled trials differ from the latter statement. Gilron et al. in Canada conducted a crossover randomized, double-blind trial of placebo, pregabalin, duloxetine, and the combination of pregabalin and duloxetine. This study was published in 2016 but initiated before the publication of the 2010 ACR criteria for FM. Therefore, they used the 1990 ACR criteria for their patients. Patients included in the study aged between 18 and 70 years, had sufficient cognitive function and language skills, and experienced pain on a daily basis for at least three months. Their exclusion criteria included, among others, major organ system disease, hypersensitivity to any of the drugs studied in this trial, mood disorders diagnosed by a psychiatrist, and history of drug abuse. Patients with uncontrolled diabetes mellitus, hypertension, narrow-angle glaucoma, or malignancies were also excluded. The primary outcome of this study was pain intensity, and the secondary outcomes included global pain relief, quality of life, sleep, and mood. This study reported insignificant differences between duloxetine and pregabalin in depressive symptoms improvement, despite duloxetine showing higher pain improvement than pregabalin [63].

Bidari et al. performed a randomized clinical trial to compare duloxetine and pregabalin for the treatment of pain and depression in women with FM in Iran from 2016 to 2017. Patients selected for this study were women aged between 18 and 65 years who did not have a history of hypersensitivity to trial medications. Patients included did not have a history of taking duloxetine, pregabalin, gabapentin, or any antidepressant, monoamine oxidase inhibitors, muscle relaxants, steroids, opioid analgesics, or benzodiazepines for certain periods of time. Patients were also excluded if they were pregnant, breastfeeding, or had intended to become pregnant during the trial. In addition, patients who had any other comorbidity that would cause chronic pain or other psychiatric conditions besides depression/anxiety disorders, chronic liver disease, renal failure, or uncontrolled narrow-angle glaucoma were excluded. Patients received either duloxetine or pregabalin, and outcomes were observed after four weeks of treatment [64]. Similarly to Gilron et al., this study did not find a significant difference in mental health or quality of life improvement between duloxetine and pregabalin and reported the superiority of duloxetine in pain improvement [63,64].

Exercise

The most effective nonpharmacologic treatments in FM management are fitness and strengthening exercises. Recommended fitness exercises can include aerobics, pool, or land-based exercises three times per week, whereas the recommendation for strengthening exercises is a one-hour session twice a week [65,66]. Evidence indicates that these types of exercises decrease pain and have a positive effect on mood and quality of life in patients with FM, in addition to improvement of endurance, function, and muscle strength [67-70].

Cognitive Behavioral Therapy

Due to the impact that depressive symptoms have on the clinical and socio-economical aspects of patients with FM, another component of its management is psychological treatment: CBT as the treatment of choice, which has shown pain relief, improvement in quality of life, and depressive symptoms, among other effects [71]. CBT generally consists of brief lectures, teaching skills, and practicing them in therapy sessions and daily life at home. Multiple skills are included. Some of them target depressive symptoms, such as cognitive reframing for mood problems and setting goals for long-term functioning [72]. CBT seems to be effective for depressive symptoms short-term while improving pain self-management and decreasing the frequency of visits to the doctor [73].

Limitations

Multiple conditions have been associated with FM, and all of them tremendously affect the management of FM and its outcome. This article review focused only on depression. Furthermore, many other aspects of FM impact patients’ overall quality of life, such as fatigue and sleep disorders. The article does not address other aspects of FM besides mood, precisely depressive symptoms. The lack of external validity due to the heterogenicity of the studies included is a major drawback of the article.

## Conclusions

As showcased by this review, the prevalence of depression in patients with FM is significantly higher than in patients without this condition. In fact, the most prevalent condition in patients previously diagnosed with FM is depression. The proven bidirectional relation between FM and depression represents important clinical implications, given that the presence of one of the diseases mentioned above increases the risk and worsens the outcome of the other. However, diagnosing depression in patients with FM represents a challenge due to overlapping symptoms and criterion contamination of assessment tools regularly applied in the screening for depression. This review of the aspects shared by FM and depression highlights how frequently we can encounter patients diagnosed with FM who developed depression. The latter may or may not be targeted already in their treatment plan. Our goal is to highlight the importance of identifying depressive symptoms in patients with FM. In addition, we aim to emphasize the positive impact that proper management of both conditions can have on patients' quality of life and course of FM. In addition to the prevalence of depression in patients with FM, we discussed the overlapping symptoms between these two conditions, mechanisms within their pathophysiology that might link them, and management strategies that effectively target and treat both diseases to improve patients’ well-being further. Despite all difficulties, proper screening of FM patients for depression is needed due to the clinical implications that depression has in patients with FM, even if the mood dysfunction remains sub-threshold. We consider that the relation between FM and depression needs further investigation to elucidate the mechanisms that connect them in order to find more effective treatments for patients presenting both conditions and avoid the cycle of depression worsening FM and vice versa.

## References

[1] D'Agnelli S, Arendt-Nielsen L, Gerra MC, Zatorri K, Boggiani L, Baciarello M, Bignami E (2019). Fibromyalgia: genetics and epigenetics insights may provide the basis for the development of diagnostic biomarkers. Mol Pain.

[2] Wolfe F (2009). Fibromyalgia wars. J Rheumatol.

[3] Shir Y, Fitzcharles MA (2009). Should rheumatologists retain ownership of fibromyalgia?. J Rheumatol.

[4] Häuser W, Fitzcharles MA (2018). Facts and myths pertaining to fibromyalgia. Dialogues Clin Neurosci.

[5] Wolfe F, Smythe HA, Yunus MB (1990). The American College of Rheumatology 1990 Criteria for the classification of fibromyalgia: report of the Multicenter Criteria Committee. Arthritis Rheum.

[6] Wolfe F, Walitt B, Perrot S, Rasker JJ, Häuser W (2018). Fibromyalgia diagnosis and biased assessment: sex, prevalence and bias. PLoS One.

[7] Jones GT, Atzeni F, Beasley M, Flüß E, Sarzi-Puttini P, Macfarlane GJ (2015). The prevalence of fibromyalgia in the general population: a comparison of the American College of Rheumatology 1990, 2010, and modified 2010 Classification Criteria. Arthritis Rheumatol.

[8] Kaltsas G, Tsiveriotis K (2022). Fibromyalgia. https://www.ncbi.nlm.nih.gov/books/NBK279092.

[9] Vargas-Alarcón G, Fragoso JM, Cruz-Robles D (2009). Association of adrenergic receptor gene polymorphisms with different fibromyalgia syndrome domains. Arthritis Rheum.

[10] Smith SB, Maixner DW, Fillingim RB (2012). Large candidate gene association study reveals genetic risk factors and therapeutic targets for fibromyalgia. Arthritis Rheum.

[11] Arnold LM, Hudson JI, Hess EV (2004). Family study of fibromyalgia. Arthritis Rheum.

[12] Haviland MG, Morton KR, Oda K, Fraser GE (2010). Traumatic experiences, major life stressors, and self-reporting a physician-given fibromyalgia diagnosis. Psychiatry Res.

[13] Hellou R, Häuser W, Brenner I (2017). Self-reported childhood maltreatment and traumatic events among Israeli patients suffering from fibromyalgia and rheumatoid arthritis. Pain Res Manag.

[14] Siracusa R, Paola RD, Cuzzocrea S, Impellizzeri D (2021). Fibromyalgia: pathogenesis, mechanisms, diagnosis and treatment options update. Int J Mol Sci.

[15] Vierck CJ Jr (2006). Mechanisms underlying development of spatially distributed chronic pain (fibromyalgia). Pain.

[16] Muir WW III, Woolf CJ (2001). Mechanisms of pain and their therapeutic implications. J Am Vet Med Assoc.

[17] Crofford LJ, Pillemer SR, Kalogeras KT (1994). Hypothalamic-pituitary-adrenal axis perturbations in patients with fibromyalgia. Arthritis Rheum.

[18] Gür A, Karakoç M, Nas K, Remzi Remzi, Cevik Cevik, Denli A, Saraç J (2002). Cytokines and depression in cases with fibromyalgia. J Rheumatol.

[19] Wolfe F, Clauw DJ, Fitzcharles MA (2016). 2016 Revisions to the 2010/2011 fibromyalgia diagnostic criteria. Semin Arthritis Rheum.

[20] Arnold LM, Bennett RM, Crofford LJ (2019). AAPT diagnostic criteria for fibromyalgia. J Pain.

[21] Erdrich S, Hawrelak JA, Myers SP, Harnett JE (2020). A systematic review of the association between fibromyalgia and functional gastrointestinal disorders. Ther Adv Gastroenterol.

[22] De Stefano R, Bruno A, Muscatello MR (2020). Oral health and fibromyalgia syndrome: a systemic review. J Funct Morphol Kinesiol.

[23] Penn IW, Chuang E, Chuang TY, Lin CL, Kao CH (2019). Bidirectional association between migraine and fibromyalgia: retrospective cohort analyses of two populations. BMJ Open.

[24] Alciati A, Sgiarovello P, Atzeni F, Sarzi-Puttini P (2012). Psychiatric problems in fibromyalgia: clinical and neurobiological links between mood disorders and fibromyalgia. Reumatismo.

[25] Ablin J, Fitzcharles MA, Buskila D, Shir Y, Sommer C, Häuser W (2013). Treatment of fibromyalgia syndrome: recommendations of recent evidence-based interdisciplinary guidelines with special emphasis on complementary and alternative therapies. Evid Based Complement Alternat Med.

[26] Macfarlane GJ, Kronisch C, Dean LE (2017). EULAR revised recommendations for the management of fibromyalgia. Ann Rheum Dis.

[27] (2022). World Health Organization: Depression. https://www.who.int/news-room/fact-sheets/detail/depression.

[28] Chang MH, Hsu JW, Huang KL (2015). Bidirectional association between depression and fibromyalgia syndrome: a nationwide longitudinal study. J Pain.

[29] Magni G, Marchetti M, Moreschi C, Merskey H, Luchini SR (1993). Chronic musculoskeletal pain and depressive symptoms in the National Health and Nutrition Examination. I. Epidemiologic follow-up study. Pain.

[30] Taloyan M, Löfvander M (2014). Depression and gender differences among younger immigrant patients on sick leave due to chronic back pain: a primary care study. Prim Health Care Res Dev.

[31] Maletic V, Raison CL (2009). Neurobiology of depression, fibromyalgia and neuropathic pain. Front Biosci (Landmark Ed).

[32] Nabeshima T, Kim HC (2013). Involvement of genetic and environmental factors in the onset of depression. Exp Neurobiol.

[33] Cohen H, Buskila D, Neumann L, Ebstein RP (2002). Confirmation of an association between fibromyalgia and serotonin transporter promoter region (5- HTTLPR) polymorphism, and relationship to anxiety-related personality traits. Arthritis Rheum.

[34] Kendler KS, Thornton LM, Gardner CO (2000). Stressful life events and previous episodes in the etiology of major depression in women: an evaluation of the "kindling" hypothesis. Am J Psychiatry.

[35] Post RM (2007). Kindling and sensitization as models for affective episode recurrence, cyclicity, and tolerance phenomena. Neurosci Biobehav Rev.

[36] Blackburn-Munro G, Blackburn-Munro RE (2001). Chronic pain, chronic stress and depression: coincidence or consequence?. J Neuroendocrinol.

[37] Ross RL, Jones KD, Ward RL, Wood LJ, Bennett RM (2010). Atypical depression is more common than melancholic in fibromyalgia: an observational cohort study. BMC Musculoskelet Disord.

[38] Gold PW, Gabry KE, Yasuda MR, Chrousos GP (2002). Divergent endocrine abnormalities in melancholic and atypical depression: clinical and pathophysiologic implications. Endocrinol Metab Clin North Am.

[39] Albrecht DS, Forsberg A, Sandström A (2019). Brain glial activation in fibromyalgia - a multi-site positron emission tomography investigation. Brain Behav Immun.

[40] Steiner J, Walter M, Gos T (2011). Severe depression is associated with increased microglial quinolinic acid in subregions of the anterior cingulate gyrus: evidence for an immune-modulated glutamatergic neurotransmission?. J Neuroinflammation.

[41] Kendler KS, Karkowski LM, Prescott CA (1999). Causal relationship between stressful life events and the onset of major depression. Am J Psychiatry.

[42] Van Houdenhove B, Egle UT (2004). Fibromyalgia: a stress disorder? Piecing the biopsychosocial puzzle together. Psychother Psychosom.

[43] Mendieta D, De la Cruz-Aguilera DL, Barrera-Villalpando MI (2016). IL-8 and IL-6 primarily mediate the inflammatory response in fibromyalgia patients. J Neuroimmunol.

[44] Wallace DJ, Linker-Israeli M, Hallegua D, Silverman S, Silver D, Weisman MH (2001). Cytokines play an aetiopathogenetic role in fibromyalgia: a hypothesis and pilot study. Rheumatology (Oxford).

[45] Cunha FQ, Lorenzetti BB, Poole S, Ferreira SH (1991). Interleukin-8 as a mediator of sympathetic pain. Br J Pharmacol.

[46] Dowlati Y, Herrmann N, Swardfager W, Liu H, Sham L, Reim EK, Lanctôt KL (2010). A meta-analysis of cytokines in major depression. Biol Psychiatry.

[47] Lanquillon S, Krieg JC, Bening-Abu-Shach U, Vedder H (2000). Cytokine production and treatment response in major depressive disorder. Neuropsychopharmacology.

[48] Tsai SJ (2021). Role of interleukin 8 in depression and other psychiatric disorders. Prog Neuropsychopharmacol Biol Psychiatry.

[49] Kleykamp BA, Ferguson MC, McNicol E (2021). The prevalence of psychiatric and chronic pain comorbidities in fibromyalgia: an ACTTION systematic review. Semin Arthritis Rheum.

[50] Løge-Hagen JS, Sæle A, Juhl C, Bech P, Stenager E, Mellentin AI (2019). Prevalence of depressive disorder among patients with fibromyalgia: systematic review and meta-analysis. J Affect Disord.

[51] Singh G, Kaul S (2018). Anxiety and depression are common in fibromyalgia patients and correlate with symptom severity score. Indian Journal of Rheumatology.

[52] Kassam A, Patten SB (2006). Major depression, fibromyalgia and labour force participation: a population-based cross-sectional study. BMC Musculoskelet Disord.

[53] Duque L, Fricchione G (2019). Fibromyalgia and its new lessons for neuropsychiatry. Med Sci Monit Basic Res.

[54] Pincus T, Williams A (1999). Models and measurements of depression in chronic pain. J Psychosom Res.

[55] de C Williams AC, Richardson PH (1993). What does the BDI measure in chronic pain?. Pain.

[56] Veltri A, Scarpellini P, Piccinni A (2012). Methodological approach to depressive symptoms in fibromyalgia patients. Clin Exp Rheumatol.

[57] Dell'Osso L, Armani A, Rucci P (2002). Measuring mood spectrum: comparison of interview (SCI-MOODS) and self-report (MOODS-SR) instruments. Compr Psychiatry.

[58] Gracely RH, Ceko M, Bushnell MC (2012). Fibromyalgia and depression. Pain Res Treat.

[59] Bernik M, Sampaio TP, Gandarela L (2013). Fibromyalgia comorbid with anxiety disorders and depression: combined medical and psychological treatment. Curr Pain Headache Rep.

[60] Marangell LB, Clauw DJ, Choy E (2011). Comparative pain and mood effects in patients with comorbid fibromyalgia and major depressive disorder: secondary analyses of four pooled randomized controlled trials of duloxetine. Pain.

[61] Lee YH, Song GG (2016). Comparative efficacy and tolerability of duloxetine, pregabalin, and milnacipran for the treatment of fibromyalgia: a Bayesian network meta-analysis of randomized controlled trials. Rheumatol Int.

[62] Häuser W, Petzke F, Sommer C (2010). Comparative efficacy and harms of duloxetine, milnacipran, and pregabalin in fibromyalgia syndrome. J Pain.

[63] Gilron I, Chaparro LE, Tu D (2016). Combination of pregabalin with duloxetine for fibromyalgia: a randomized controlled trial. Pain.

[64] Bidari A, Moazen-Zadeh E, Ghavidel-Parsa B, Rahmani S, Hosseini S, Hassankhani A (2019). Comparing duloxetine and pregabalin for treatment of pain and depression in women with fibromyalgia: an open-label randomized clinical trial. Daru.

[65] Brosseau L, Wells GA, Tugwell P (2008). Ottawa Panel evidence-based clinical practice guidelines for aerobic fitness exercises in the management of fibromyalgia: part 1. Phys Ther.

[66] Brosseau L, Wells GA, Tugwell P (2008). Ottawa Panel evidence-based clinical practice guidelines for strengthening exercises in the management of fibromyalgia: part 2. Phys Ther.

[67] Meiworm L, Jakob E, Walker UA, Peter HH, Keul J (2000). Patients with fibromyalgia benefit from aerobic endurance exercise. Clin Rheumatol.

[68] Gowans SE, deHueck A, Voss S, Silaj A, Abbey SE, Reynolds WJ (2001). Effect of a randomized, controlled trial of exercise on mood and physical function in individuals with fibromyalgia. Arthritis Rheum.

[69] Gusi N, Tomas-Carus P, Häkkinen A, Häkkinen K, Ortega-Alonso A (2006). Exercise in waist-high warm water decreases pain and improves health-related quality of life and strength in the lower extremities in women with fibromyalgia. Arthritis Rheum.

[70] Sosa-Reina MD, Nunez-Nagy S, Gallego-Izquierdo T, Pecos-Martín D, Monserrat J, Álvarez-Mon M (2017). Effectiveness of therapeutic exercise in fibromyalgia syndrome: a systematic review and meta-analysis of randomized clinical trials. Biomed Res Int.

[71] Bernardy K, Klose P, Welsch P, Häuser W (2018). Efficacy, acceptability and safety of cognitive behavioural therapies in fibromyalgia syndrome - A systematic review and meta-analysis of randomized controlled trials. Eur J Pain.

[72] Lumley MA, Schubiner H, Lockhart NA, Kidwell KM, Harte SE, Clauw DJ, Williams DA (2017). Emotional awareness and expression therapy, cognitive behavioral therapy, and education for fibromyalgia: a cluster-randomized controlled trial. Pain.

[73] Minelli A, Vaona A (2012). Effectiveness of cognitive behavioral therapy in the treatment of fibromyalgia syndrome: a meta-analytic literature review. Reumatismo.

